# Neural Crest Stem Cells in Juvenile Angiofibromas

**DOI:** 10.3390/ijms23041932

**Published:** 2022-02-09

**Authors:** Bernhard Schick, Lukas Pillong, Gentiana Wenzel, Silke Wemmert

**Affiliations:** Department of Otolaryngology, Saarland University Medical Center, 66421 Homburg, Germany; bernhard.schick@uks.eu (B.S.); lukas.pillong@uks.eu (L.P.); gentiana.wenzel@uks.eu (G.W.)

**Keywords:** juvenile angiofibroma, neural crest stem cell, CD271, epithelial-mesenchymal transition, MMP3

## Abstract

The etiology of juvenile angiofibroma (JA) has been a controversial topic for more than 160 years. Numerous theories have been proposed to explain this rare benign neoplasm arising predominately in adolescent males, focusing mainly on either the vascular or fibrous component. To assess our hypothesis of JA’s being a malformation arising from neural crest cells/remnants of the first branchial arch plexus, we performed immunohistochemical analyses of neural crest stem cells (NCSC) and epithelial-mesenchymal transition (EMT) candidates. Immunoexpression of the NCSC marker CD271^p75^ was observed in all investigated JA’s (*n* = 22), mainly around the pathological vessels. Close to CD271^p75^-positive cells, high MMP3-staining was also observed. Additionally, from one JA with sufficient material, RT-qPCR identified differences in the expression pattern of PDGFRβ, MMP2 and MMP3 in MACS^®^-separated CD271^p75^positive vs. CD271^p75^ negative cell fractions. Our results, together with the consideration of the literature, provide evidence that JA’s represent a malformation within the first branchial arch artery/plexus remnants deriving from NCSC. This theory would explain the typical site of tumor origin as well as the characteristic tumor blood supply, whereas the process of EMT provides an explanation for the vascular and fibrous tumor component.

## 1. Introduction

Juvenile angiofibroma (JA) is a rare tumor accounting for 0.05% of head and neck neoplasms [1]. It presents with four typical features: (1) fibrovascular architecture with irregular lined vessels embedded in a variable fibrous setting, (2) typical tumor localization in the pterygoid fossa/posterior nasal cavity close to the sphenopalatine foramen/pterygoid base with extension into the nasopharynx, (3) blood supply mainly from the external but also from the internal carotid artery, and (4) presentation typically in adolescent males [1]. Current theories focus mainly on the vascular or fibrous tumor components [1,2], not completely explaining its combined fibrovascular characteristics.

Pivotal studies in understanding JA’s biology were descriptions of frequent β-catenin mutations; studies focusing on members of the Wnt-signalling pathway [3,4] as well as descriptions of genetic changes [5,6,7,8,9,10]. Further studies focused on angiogenesis or various growth factors as well as sex hormones and their receptors [1,11,12,13,14] being involved in JA’s. Proliferation was noted either in the vascular or fibrous component [12,13,14].

Additionally, the anatomical tumor origin is still widely discussed as well. Based on clinical and radiological findings, the pterygopalatine fossa, the sphenopalatine foramen, the vidian canal, the choane/nasopharynx, and the pterygoid wedge have been proposed as possible tumor origins [1,15,16]. These different structural abnormalities need to be considered for preoperative assessment since surgical resection is the primary therapeutic option [17,18]. Despite all these reports the etiology, the tumor cell of origin, and the anatomical site are still not successfully defined.

We propose and discuss herein, based on our experimental findings, that the neural crest stem cells (remnants of the first branchial arch artery plexus) are the tumor cells of origin in JA’s.

## 2. Results

### 2.1. Sample Characterization

**Figure 1:**
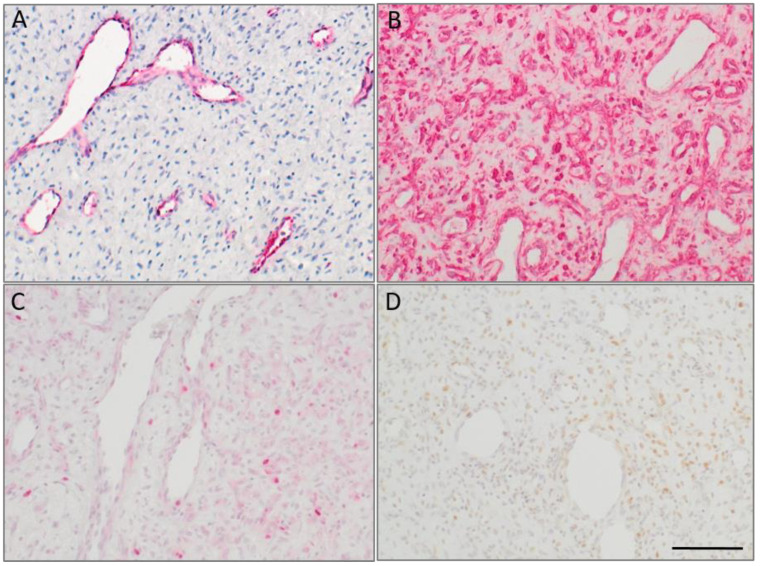
Immunohistochemical characterization of typical features of JA. (**A**) Positive staining of endothelial cells (CD31), (**B**) Vimentin, (**C**) Ki67, (**D**) PCNA; scale bar 100 µm. Counterstaining was performed with hematoxylin.

In the investigated JA’s (*n* = 22), the vessels demonstrated specific immunohistochemical staining for CD31 (score 3), whereas the surrounding stromal cells were positive for Vimentin (score 3). These results reflect the characteristic fibrovascular architecture in all specimens (Figure 1) and are in line with the previous histopathological JA’s classification. Patients’ characteristics and immunohistochemical results are listed in Table 1.

To determine if enhanced proliferation was suitable to obtain a hint as to where the tumor cell of origin is placed, we performed Ki67-staining and observed single positive signals in vascular and also fibrous components (*n* = 22, score 0–1). The marker PCNA demonstrated increased positive staining across tumors (*n* = 10, score 3) that were not limited to the vascular or fibrous components.

### 2.2. CD271^p75^ Analysis

**Figure 2:**
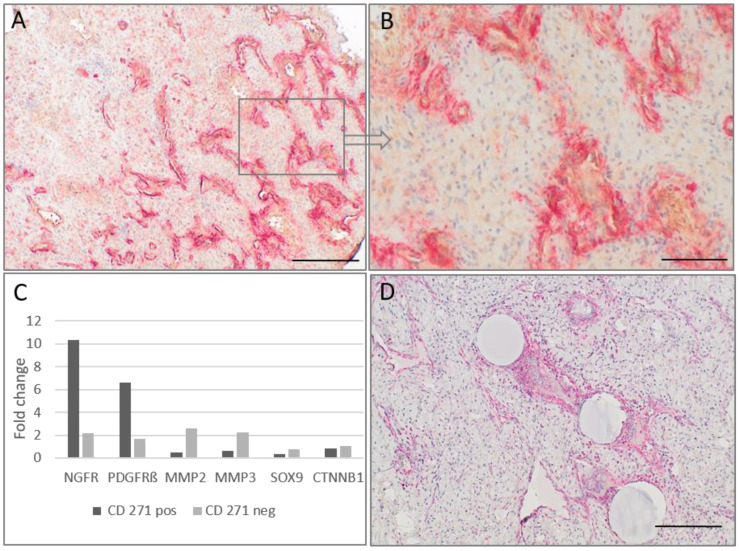
(**A**,**B**) Immunochemical staining of CD271^p75^ (Fast red) and PDGFRβ (DAB); scale bar, 500 µm (**A**) and 100 µm (**B**), (**C**) Quantitative real time PCR expression of CD271^p75^positive vs. CD271^p75^negative cells in one JA, (**D**) Immunochemical staining of MMP3 (Fast red); scale bar, 200 µm.

In accordance to our embryological thoughts we looked for CD271^p75^-positive cells in JA’s as possible persisting neural crest cells. We used CD271-staining and detected in all analyzed JA’s (*n* = 22) CD271^p75^positive cells. Interestingly, the tumors showed a heterogeneous staining pattern. In areas with a high percentage of pathological vessels, an intense CD271^p75^-staining was found along the irregularly configured endothelial lined vessels close to the endothelium, whereas in sections with predominantly fibrous tumor components only small amounts of or no CD271^p75^-staining was observed (Figure 2). Additionally, interspersed single stromal cells positive for the neural crest marker HNK1/CD57 were found (*n* = 11).

In one case with fresh tumor tissue available, we were able to sort between CD271^p75^positive and CD271^p75^negative JA cells. RNA was extracted and a Human Mesenchymal Stem Cell RT^2^ Profiler™ PCR Array was used for expression analyses. In the CD271^p75^positive cells a more than 2-fold overexpression of ACTA2, BMP4, FGF10, FUT1, HGF, JAG1, MCAM, NES, NGFR (CD271^p75^), PDGFRβ, PPARG, SOX9, VAM1, and WNT3A was found. Furthermore, BMP7, COL1A1, CSF3, CTNNB1, IFG1, MMP2, POU5F1, RUNX2 were underexpressed in CD271^p75^positive cells compared with CD271^p75^negative cells.

Relative mRNA expression of NGFR (CD271^p75^), PDGFRβ, SOX9, CTNNB1, MMP2 and MMP3 in isolated CD271^p75^positive and CD271^p75^negative cell fractions were determined and normalized to β2M. The real-time PCR validated the RT^2^ Profiler™ PCR Array analysis of NGFR (CD271^p75^) and PDGFRβ in isolated CD271^p75^positive cell fractions. CTNNB1 and MMP2 were detected in isolated CD271^p75^negative fractioned cells; however, SOX9 was not detected. Additionally, an elevated level of MMP3 was observed in the CD271^p75^negative cell fraction as well (Figure 2C).

### 2.3. Matrix Metalloproteinase-3 (MMP3) and Epithelial-Mesenchymal Transition (EMT)

**Figure 3:**
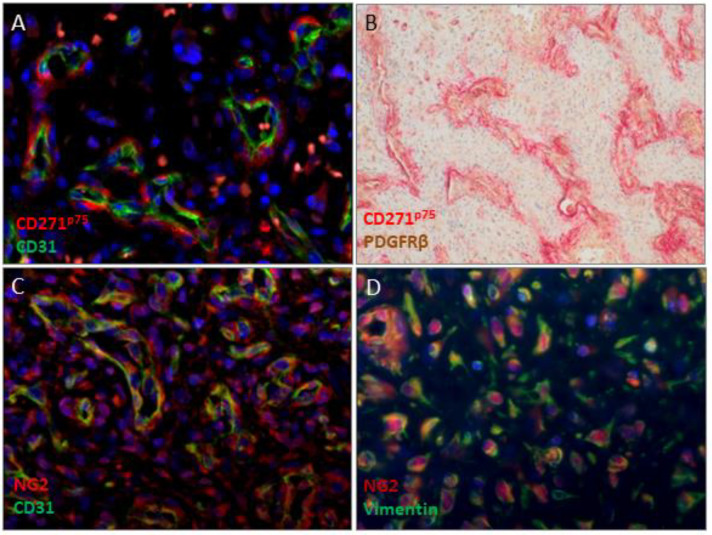
Expression patterns of EMT. (**A**,**C**,**D**) Immunofluorescence and (**B**) Immunohistochemistry of juvenile angiofibromas. (**A**) Immunostaining of CD271^p75^ (Alexa-568, red) and CD31 (Alexa-488, green), (**B**) Immunostaining of CD271^p75^ (Fast red) and PDGFRβ (DAB), (**C**) Immunostaining of NG2 (red) and CD31 (green), (**D**) Immunostaining of NG2 (red) and Vimentin (green). Immunofluorescent sections were counterstained with Hoechst 33342, immunohistochemical sections were counterstained with hematoxylin. Magnification (**A**,**C**,**D**) ×40; magnification (**B**) ×10.

Our assumption of persisting neural crest cells only along the pathological vessels was not sufficient to also explain the fibrous tumor component. Moreover, CD271^p75^positive cells were not the dominant cell type in our specimens, but this likely makes them a possible starting point for tumor development. MMP3 expression is an early step of EMT and is found in vascular pathologies [19]. Since our data provides evidence of increased MMP3 expression in CD271^p75^negative cells, we investigated MMP3 in more detail. High MMP3-staining next to the pathological tumor vessels and in the fibrovascular areas was observed (Figure 2D). Additionally, analysis of PDGFRβ and NG2 as pericyte markers revealed in all analyzable patients (*n* = 21) a positive PDGFRβ-staining with different amounts of positive cells in the tumors. NG2 immunostaining also demonstrated very heterogeneous findings ranging from completely negative tumor areas up to a strong positive stromal staining (Figure 3).

## 3. Discussion

Juvenile angiofibromas (JA) are histopathologically benign and highly vascular neoplasms mainly occurring in young adolescent men. The etiology of this rare tumor has been a controversial topic over the years. Whereas some researchers believe it is a genuine tumor, others consider it is the result of vascular malformations caused by non-absorption of artery residues within the first branchial arch during development. Hormones and genetic effects are related to its etiology as well. It was previously thought that JA’s originate from the nasopharynx. However, several studies emphasized that it originated from the upper lip of the sphenopalatine foramen at the junction of the sphenoid process of the palate and the pterygoid process. Others considered that it originated from the pterygoid canal [1].

Here, we present a novel theory regarding the JA’s etiology that explains, to our knowledge for the first time in literature, the characteristic features of these tumors.

**Figure 4:**
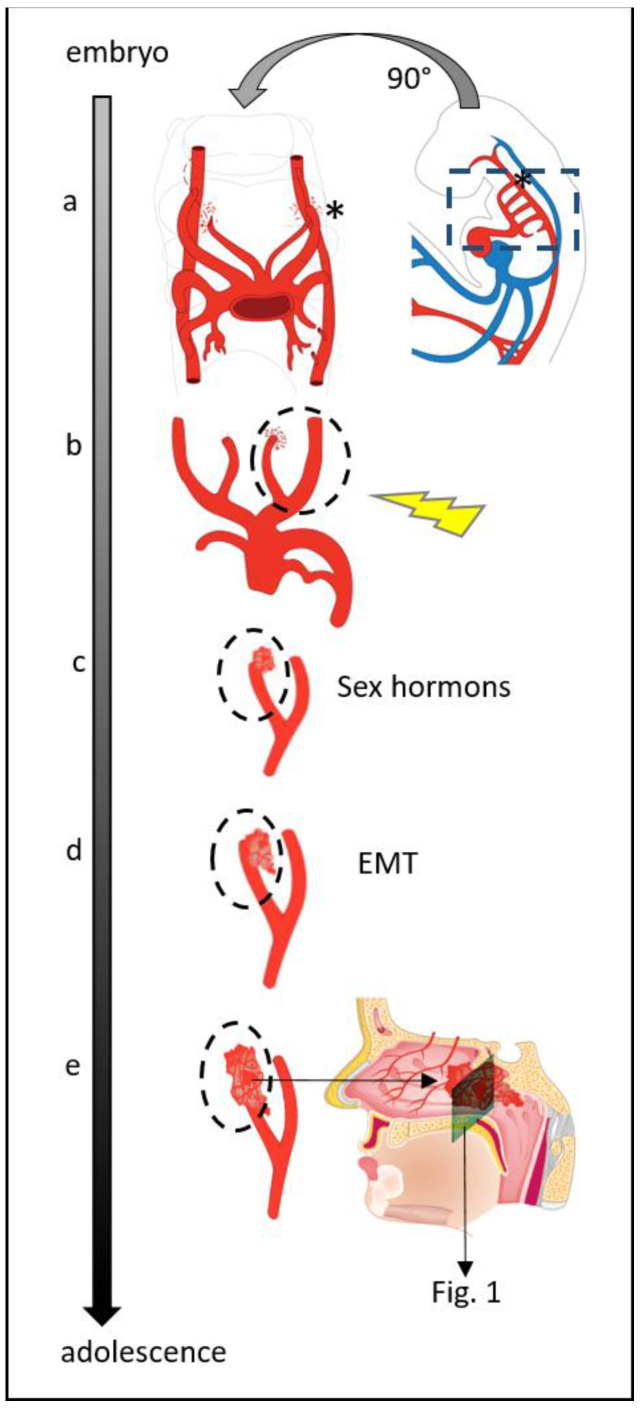
(**a**) The first branchial arch artery is important in embryology to ensure the blood supply of the brain. This vascular structure resolves and the carotid artery arises in a later developmental stage. (**b**) The temporarily present first branchial arch artery recedes forming a vascular plexus. Vascular spaces may persist in the area of the sphenopalatine artery branches due to unknown factors (yellow arrow) and (**c**) these start to proliferate under a hormonal stimulus. (**d**) EMT starts within these pathological vessels and the tumor size increases forming the fibrous tumor component. (**e**) The typical location of the tumor and its blood supply are therefore within the nasopharyngeal area.

We hypothesize that the neural crest stem cells (remnants of the first branchial arch artery plexus) are the tumor cells of origin in JA’s developed as a result of unknown disturbances in first branchial arch regression (Figure 4). This theory explains their site of origin and their typical blood supply.

The craniofacial development represents a complex process that can be possibly disturbed at several steps. It is known that one third of all human congenital defects are craniofacial anomalies [20]. An embryological origin is already described for chordoma: an incomplete embryological cell regression (atavism) is considered its known starting point [21]. This idea fits well into a previous study on 32 JA’s, in which they are defined as a vascular malformation rather than a real tumor [22].

The neural crest stem cells play a pivotal role in the craniofacial development. They migrate, besides many other locations, into the branchial arches and are involved in vascular development [20,23]. In the branchial arches the cranial NCSCs are known to be involved in the remodeling process of the pharyngeal arteries [24]. Neural crest stem cells, that have not undergone their natural fate and are still present, may be stimulated at a later stage through an undefined trigger and/or hormone influence and develop into JA. The neural crest stem cell marker CD271 was detected in our study along the typical tumor vessels of JA’s. This finding fits to a previous study which shows Laminin α2 beside the embryological vessels of the brain area but also in JA`s, indicating the presence of early developmental stage vessels in these tumors [16]. Additionally, an embryological collagen Iα1/Iα2-ratio and the TSHZ1-protein, which is related to the first and second branchial arch, have been observed in JA’s in previous studies [25,26].

The postulated development from neural crest stem cells attributed to the first branchial arch artery can also explain the typical location and blood supply of JA’s. Between days 22–24 of embryological development, a temporary vascular blood supply for the developing brain is needed by the first branchial arch artery. This vessel transforms later into a plexus that partially resolves and is incorporated in the formation of the maxillary artery branches [2,27]. Harrison [28] has already observed in fetal samples endothelial lined spaces around the area of the sphenopalatine foramen. We assume them to be remnants of the first branchial arch plexus (Figure 4). Thus, persisting neural crest stem cells of the first branchial arch would explain the localization of JA’s in the distal area of the maxillary/sphenopalatine branches and the blood supply derived from the internal carotid artery.

Besides the typical findings of vascular irregularities, the fibrous tumor component present in JA’s can be explained through EMT, a process which plays an important role in embryology and in many other fields (e.g., wound healing, stem cell differentiation, fibrosis and tumor pathology) [29]. In accordance with previous reports, our findings support that EMT in JA’s is the building-up of connective tissue as part of their natural developmental process [20]. Additionally, Wnt-, FGF- and TGFβ-family members have been reported to be expressed in JA’s [1] but are also able to regulate neural crest delamination and act as inducers of EMT in development [29,30]. Moreover, it has been reported that NCSCs affected by PDGFRβ abnormally differentiate into smooth muscle cells at the first and second pharyngeal arch arteries [27]. Overexpression of PDGF has been found in JA’s in previous studies as well [31,32], and the observation of smooth muscle actin in JA’s [11] may reflect a pathological change inducing smooth muscle expression in NCSCs that undergo further mesenchymal differentiation. In addition, hepatocyte growth factor, Insulin-like growth factor and VEGF are further candidates upregulated in JA’s and are described as inducers of EMT [9,29].

EMT is also characterized by loss of E-cadherin and increased expression of N-cadherin [28]. This is in accordance to our previous study which demonstrated that 8 out of the 13 analyzed JA’s exhibited a strong N-cadherin staining, whereas E-cadherin was absent [33].

MMP3 is known to be an inducer of EMT [28,34] and MMP3 polymorphism contributes to heterogeneity in vascular remodeling, resulting in cerebrovascular diseases [19]. We present herein extensive MMP3 expression along the pathological tumor vessels. Additionally, increased MMP2 and MMP9 have been described as part of EMT and were reported in JA’s before [35]. Furthermore, increased integrin-expressions, also expected in EMT [29], have been observed for α1 and β1-integrin expressions in JA’s previously [36] and Vimentin, a marker for final mesenchymal differentiation in EMT [29], is expressed strongly in stromal cells of JA’s [13]. Interestingly, Pauli et al. [37] reported stromal cells in JA’s presenting still ongoing endothelial differentiation, proving cellular plasticity at endothelial and mesenchymal level. In our previous cell culture study of fibrous cells derived from JA’s, mesenchymal-epithelial transition could be observed in single cells, indicating the possibility that EMT has a reversible nature [38].

Considering the heterogeneous composition of these tumors, bulk-cell analyses are hardly able to resolve differences in gene expression deriving from differential cell populations rather than from differences in tumor cells. This can lead to discordant results when comparing transcriptional expression levels with the immunoreactivity restricted to the endothelial and stromal cells observed in the tissue. In one JA, fresh tumor material was available for cell sorting, and RT PCR was investigated to identify differential expression genes (DEGs) in the different cell fractions. As expected, the CD271^p75^positive NCSCs exhibited a significant upregulation of the marker MCAM, NES, NGFR (CD271^p75^) and PDGFRβ, whereas the negative cell fraction showed alterations of EMT components, e.g., BMP7, COL1A1, MMP2 and MMP3.

Additionally, based on the presence of EMT in JA’s, two further findings can be explained: First, it might be the natural fate of the first branchial arch plexus remnants to undergo EMT and to dissolve. This would give an explanation that in rare cases, spontaneous regression was observed in JA’s [39,40]. Second, even though many studies on hormones and hormone receptors have been performed in the past [11,12,41], no convincing answer for the cause of the male predominance in JA’s has been given so far. However, sex hormone influence was shown to play a role in vascular angiogenesis [42,43]. Changes in Endothelin-1 (Edn1) and its receptor type-A (Edn1/Ednra) pathway result in malformation of pharyngeal arch-derived craniofacial structures as well as thoracic arteries in mice [26]. Additionally, the effect of cellular Edn1 can be altered by the influence of estrogen. Thus, estrogen might be able to block the pathological Edn1 effect on the vessels in JA’s. Moreover, the detected β-catenin changes in JA’s [3,4] must be taken into account since its accumulation increases the androgen sensitivity of JA’s.

## 4. Materials and Methods

### 4.1. Tumor Specimens

Twenty-four formalin fixed, paraffin embedded tissue samples (FFPE) of 22 male patients, age 1 to 46 years (average 14 years), operated on and diagnosed at Saarland University Medical Centre and samples from our partners in Heidelberg (Germany), Erlangen (Germany), Aachen (Germany) and Lublin (Poland) between 1998–2020, were analyzed in this study. In one case, fresh specimens were available for analysis as well. The tumor stage ranged from 1 to 4 [44]. Written informed consent has been obtained from all patients and use of the human tissues had been performed according the Code of Ethics of the World Medical Association (Declaration of Helsinki) as well as approved by the Institutional Review Board (#218/10) at the Saarland University.

### 4.2. Immunohistochemistry

To detect the expression of neural crest cells and markers related to epithelial-mesenchymal transition (EMT) in JA’s, FFPE samples (*n* = 22) were utilized. From each sample 3 micron slides were deparaffinized and rehydrated, and antigen retrieval was performed using heat induced epitopes retrieval (HIER) cooked in a citrate buffer (pH 6.0). Incubation with the specific primary antibodies CD271^p75^, MMP3, CD31, Vimentin, NG2, HNK1 and PDGFRβ was performed over night at 4 °C. The dilutions and suppliers of primary antibodies for IHC are listed in Table 2. For negative controls, antibody dilution buffer without primary antibodies was applied. The DAKO Fast Red and DAB Kits (K5005 and K5007, Dako, Glostrup, Denmark) were used for detection according to the manufacturer’s instructions. Afterwards, the slides were counterstained with Mayer’s Hematoxylin (MHS32, Merck, Darmstadt, Germany). Immunofluorescence was performed with Alexa-Fluor 488 and Alexa-Fluor 568 (Thermo Fisher Scientific, Dreieich, Germany), counterstained with Hoechst 33342 (Thermo Fisher Scientific, Dreieich, Germany) and visualized on an Olympus BX61 microscope (Olympus, Hamburg, Germany). The percentage and distribution of positive cells was estimated in stromal cells and endothelial cells of JA’s by two independent observers. The overall immunoreactivity was scored in a 4-tier scale system based on the percentage of positive cells: 0, negative; 1, immunoreactivity in less than 25% of the cells; 2, moderate immunoreactivity in less than 75% of the cells; and 3, immunoreactivity in 75% or more of the cells.

### 4.3. Magnetic-Activated Cell Sorting (MACS) of CD271^p75^positive and CD271^p75^negative Cells

Single-cell suspensions from fresh angiofibroma tissue (*n* = 1) were obtained using the Whole Skin Dissociation Kit (Miltenyi Biotec, Bergisch Gladbach, Germany) without enzyme P. After dissociation, Red Blood Cell Lysis Solution (Miltenyi Biotec, Bergisch Gladbach, Germany) and Dead Cell Removal Kit (Miltenyi Biotec, Bergisch Gladbach, Germany) were performed before using MACS^®^ with the CD271 MicroBead Kit (Miltenyi Biotec, Bergisch Gladbach, Germany) according the manufacturer’s protocols. Total RNA of CD271^p75^positive and CD271^p75^negative cell fractions were isolated with the RNeasy Micro Kit (Qiagen, Hilden, Germany) with on-column DNAse treatment, and were quantified by NanoDrop (Thermo Fisher Scientific, Dreieich, Germany).

### 4.4. Real-Time Polymerase Chain Reaction (RT-PCR) Based Array Analysis

A Human Mesenchymal Stem Cell RT² Profiler™ PCR Array (PAHS-082Z, Qiagen, Hilden, Germany) was used to screen a panel of 84 genes representative of stemness and mesenchymal stem cell-specific markers in the CD271^p75^positive and CD271^p75^negative JA’s cells of one patient from whom sufficient RNA was available. 400 ng total RNA was reverse transcribed into cDNA using the RT² First Strand Kit (PAHS-082Z, Qiagen, Hilden, Germany). The Human Mesenchymal Stem Cell RT^2^ Profiler™ PCR Array was performed according to the manufacturer’s instructions in combination with RT² SYBR^®^Green qPCR Mastermix using an ABI StepOne Plus™ instrument (Applied Biosystems, Foster City, CA, USA) under the following conditions: 95 °C for 10 min, then 40 cycles at 95 °C for 15 s, and 60 °C for 1 min. On Array housekeeping genes (ACTB, β2M) were used for normalization of the sample data by calculating the ΔCt for each gene of interest in the plate, and fold changes of gene expression were analyzed and generated by using the GeneGlobe Data Analysis Center (https://geneglobe.qiagen.com/us/analyze, accessed on 26 November 2021).

### 4.5. Real-Time PCR

To validate the gene expression changes of interest, the real-time PCR was used. 50 ng total RNA of CD271^p75^positive and CD271^p75^negative sorted cells were reverse transcribed using Superscript IV Vilo kit and TaqMan^®^ assays (Thermo Fisher Scientific, Dreieich, Germany) were performed. In addition, a no template control was performed and quantitative real-time PCR reactions were executed in technical triplicates for each primer pair (Table 3) using an ABI StepOne Plus™ instrument. The thermal profile for qPCR was 20 s pre-incubation at 95 °C for one cycle, followed by 40 cycles of 95 °C for 1 s, and 60 °C for 20 s. For relative quantification, fold change for targeted genes was normalized to the houskeeping gene β2M according to the 2^−∆∆CT^ method.

## 5. Conclusions

We herein provide evidence that JA’s represent a malformation within the first branchial arch artery/plexus remnants and derive from neural crest stem cells. Our study offers an explanation for the typical site of tumor origin in the area of the sphenopalatine artery branches and the characteristic tumor blood supply. Additionally, the process of EMT can explain the vascular as well as the fibrous tumor component. Their male predominance might occur when hormones act directly on angiogenesis in the pathological vessels. Persisting neural crest stem cells seem to be the tumor cell of origin and their detection could be used to confirm the diagnosis of JA’s. Although our proposed etiology cannot account for all molecular pathologies of JA’s, it gives us a more detailed explanation regarding the development of this fascinating tumor. Additionally, larger studies are needed for a better understanding of the crosstalk between the different cells and how these alterations modulate tumor growth and angiogenesis.

## Figures and Tables

**Table 1:** Patient characteristics and immunohistochemical results.

Case	Grading	Age	Vimentin	CD31	CD271	PDGFRß	MMP3	NG2	PCNA	Ki67	HNK1
1	3a	13	3	3	2	2	1–2	1–2	2	-	-
1a	rec	15	-	3	2	2	1–2	0–1	-	1	-
2	rec	15	3	3	2	2	2	2	2	1	1
3	3a	12	3	3	1	2	3	1	2	1	-
4	1	15	3	3	2	2	2–3	1	-	1	-
5	unk	17	3	3	2	1	2	3	-	1	-
6	3a	11	3	3	2	2	3	2	2	1	-
7	3b	14	3	3	1	2	3	2	2	2	1
8	rec	18	-	-	2	2	3	-	-	-	-
8a	rec	19	3	3	2	2	3	-	2	1	1
9	rec	13	3	3	2	2	2	2	2	3	1
10	unk	17	3	3	1	1	2–3	2	2	1	1
11	3a	17	3	3	3	3	2–3	1	2	2	0–1
12	4	13	3	3	2	2	1	0–1	-	0–1	1
13	2	18	3	3	1	1	2	1	-	0	-
14	3a	12	3	3	2	2	3	2	-	-	1
15	unk	20	3	3	3	2	3	2	-	-	1
16	unk	24	3	3	2	2	3	1–2	-	-	-
17 *	unk	20	3	3	3	3	3	-	-	-	1
18	unk	13	-	3	2	3	3	0	-	-	1
19	3a	21	3	3	1	-	-	2	-	2	-
20	3b rec	46	3	3	2	2	2	0	-	1	-
21	3a	unk	3	3	2	1	1	0	-	0	-
22	unk	17	3	3	1	2	1	-	-	0	-

*—Patient with sufficient material for MACS cell sorting and RT PCR; unk-unknown; rec-recurrence.

**Table 2:** Primary antibodies for Immunohistochemistry.

Primary Antibody	Concentration	Reference No, Company
CD271^p75^-NGFR	1:1000	MA5-31968, Thermo Fisher Scientific, Dreieich, Germany
MMP3	1:300	ab52915, Abcam, Cambridge, UK
CD31	1:40	M0823; Dako Agilent, Santa Clara, CA, USA
Vimentin	1:700	M0725, Dako Agilent, Santa Clara, CA, USA
NG2	1:500	ab129051, Abcam, Cambridge, UK
PDGFRβ	1:1000	TA506230, Thermo Fisher Scientific, Dreieich, Germany
HNK1 (CD57)	1:100	MA5-11605, Thermo Fisher Scientific, Dreieich, Germany
Ki67	1:50	ab833, Abcam, Cambridge, UK
PCNA	1:10.000	ab29, Abcam, Cambridge, UK

**Table 3:** TaqMan assays for qPCR.

Gene	RefSeq Accession	Sequence
CTNNB1	NM_001098209.1	Hs00355045_m1
MMP2	NM_001127891.3	Hs00234422_m1
MMP3	NM_002422.4	Hs00968305_m1
NGFR (p75)	NM_002507.3	Hs00609976_m1
PDGFRβ	NM_002609.3	Hs01019589_m1
SOX9	NM_000346.3	Hs00165814_m1

## Data Availability

Data is contained within the article.
